# Decoupling metabolic defense: Mutational and recombinational chaos in Sod1-deficient cells

**DOI:** 10.1371/journal.pgen.1012326

**Published:** 2026-10-05

**Authors:** Yang Sui, Dao-Qiong Zheng, Yeke Wang, Shan-Shan Zhao, Ke-Jing Li, Cunqi Ye, Ke Zhang

**Affiliations:** 1 National Key Laboratory of Biobased Transportation Fuel Technology, Ocean College, Zhejiang University, Zhoushan, China; 2 Hainan Institute, Zhejiang University, Sanya, China; 3 College of Life Sciences, Zhejiang University, Hangzhou, China; University of Georgia, UNITED STATES OF AMERICA

## Abstract

Superoxide dismutase 1 (Sod1) is long recognized as a frontline antioxidant, yet its broader role in safeguarding the eukaryotic genome remains under-explored. Here, using a yeast model, we reveal that Sod1 is an indispensable guardian of genomic integrity. Its absence unleashes a genomic storm: whole-genome sequencing uncovered a dramatic surge in single nucleotide variations (13-fold), loss of heterozygosity (12-fold), chromosomal rearrangements (4-fold), and a staggering 48-fold increase in aneuploidy. Remarkably, this genomic instability was completely abolished under anaerobic conditions, identifying reactive oxygen species as the sole executioner. We demonstrate that Sod1 deficiency sculpts a unique mutational landscape dominated by C:G to A:T transversions—a molecular fingerprint of 8-oxoguanine overproduction. Mechanistically, these lesions are exacerbated by error-prone DNA polymerase ζ but mitigated by the concerted actions of Ogg1-mediated excision and DNA polymerase η. Critically, we show that Amyotrophic Lateral Sclerosis-associated variants are not created equal: the H48Q mutation shatters genome stability, whereas others (A4V, G36R, and G93A) remain benign in this context. Unlike yeast Sod1, human Sod1 is highly resistant to amino acid substitutions outside copper-binding sites, thus preventing the loss of enzyme function. Overall, our findings provide a mechanistically framework for understanding how Sod1 dysfunction drives genome instability in eukaryotic cells.

## Author summary

Sod1 is a primary antioxidant defense enzyme, yet its comprehensive role in preserving eukaryotic genome stability remains insufficiently mapped. Using a diploid yeast model, we demonstrate that Sod1 loss triggers global genomic chaos. Anaerobic growth and antioxidative agent abolish this instability, confirming reactive oxygen species as the sole genotoxic driver. Mechanistically, Sod1 deficiency drives a distinct mutational spectrum dominated by C:G to A:T transversions resulting from 8-oxoguanine accumulation. While the error-prone DNA polymerase zeta (Rev3) promotes these mutations, Ogg1-mediated base excision repair and DNA polymerase eta (Rad30) actively suppress them. Crucially, evaluation of clinical Amyotrophic Lateral Sclerosis (ALS)-associated *SOD1* mutations reveals functional heterogeneity: the metal-binding H48Q variant severely shatters genome stability, whereas variants such as A4V, G36R, and G93A remain benign in this context. Overall, this study establishes a high-resolution framework illustrating how antioxidant loss drives global genome instability and provides mechanistic insights into Sod1-linked human pathologies.

## Introduction

To mitigate the deleterious effects of reactive oxygen species (ROS), aerobic organisms have evolved a sophisticated antioxidant defense network, in which superoxide dismutase (Sod) plays a pivotal role [1]. Sod enzymes catalyze the disproportionation of highly reactive superoxide radicals (O_2_•^-^) into molecular oxygen (O_2_) and hydrogen peroxide (H_2_O_2_) [2]. The resulting H_2_O_2_ is subsequently detoxified into water by downstream enzymes, such as catalase and glutathione peroxidase, providing a comprehensive barrier against oxidative damage. In mammals, three distinct Sod (1–3) isoforms are present. Sod1 is a Cu^2+^/Zn^2+^ enzyme found predominantly in the cytosol, mitochondrial intermembrane space, and nucleus. Sod2, a Mn^2+^ enzyme, is located in the mitochondrial matrix. Sod3, another Cu^2+^/Zn^2+^ enzyme, is extracellular. Among these, Sod1 has garnered significant attention due to its prominent role in human pathologies, most notably Amyotrophic Lateral Sclerosis (ALS) [3–5]. Specific mutations in the *SOD1* gene (e.g., D90A, A4V, and G93A) are primary drivers of familial ALS (fALS) [6]. Beyond neurodegeneration, Sod1 dysfunction is increasingly implicated in oncology [7]. While elevated *SOD1* expression correlates with poor prognosis and cancer progression in certain lineages [8]*, SOD1* knockout mice are predisposed to late-onset liver tumors, suggesting that Sod1 deficiency may also drive tumorigenesis [9]. This duality underscores the role of Sod1 as a context-dependent regulator of cellular homeostasis.

The budding yeast *Saccharomyces cerevisiae* is an established model for elucidating the genetic and physiological functions of Sod1. In yeast, *sod1*Δ mutants exhibit characteristic auxotrophy for lysine and methionine, which can be rescued by augmenting intracellular NADPH or glutathione levels [10,11]. Beyond its canonical antioxidant role, Sod1 is essential for sporulation [12] and undergoes Mec1/ATM-dependent nuclear translocation upon H_2_O_2_ exposure to protect the genome [13]. Recent studies have utilized yeast to model ALS-linked mutations, revealing that these variants impair vacuolar acidification, perturb metabolic regulation, and accelerate senescence [14]. Furthermore, Sod1 deficiency has been shown to exacerbate genomic instability in *rad51*Δ backgrounds, indicating significant crosstalk between oxidative stress responses and double-strand break (DSB) repair [15]. While previous studies using genetic reporter genes have identified elevated DNA mutation and recombination rates in *sod1*Δ strains [16–18], a comprehensive, genome-wide map of the mutational consequences of Sod1 loss has yet to be established.

In the present study, we utilized mutation accumulation (MA) experiments coupled with whole-genome sequencing to systematically evaluate the global impact of Sod1 deficiency on genome integrity in a heterozygous diploid yeast model. Our results reveal that *sod1* deletion triggers profound genomic instability, characterized by elevated rates of point mutations, chromosomal rearrangements, and aneuploidy. We further delineate how base excision repair (BER) and translesion synthesis (TLS) pathways shape these distinct mutational signatures. Finally, we characterize the genotoxic effects of several fALS-associated *SOD1* variants. Together, these findings provide a high-resolution view of how Sod1 maintains genomic stability and offer mechanistic insights into diseases associated with Sod1 dysfunction.

## Results

### Sod1 deficiency induces oxidative stress and genomic instability in yeast model

To investigate the phenotypic and genetic consequences of Sod1 deficiency, we deleted the *SOD1* gene in two haploid *S. cerevisiae* strains, SY165 and SY166, which are isogenic to W303-1A and YJM789, respectively (Table 1). Mating of these haploids yielded the diploid *sod1*Δ/Δ mutant, designated as WYsod1. As shown in Fig 1A, the WYsod1 strain exhibited restricted growth on standard rich medium (YPD) and heightened sensitivity to H_2_O_2_, paraquat, and menadione compared to the wild-type control. Notably, exogenous supplementation with 5 mM glutathione (GSH), a potent antioxidant, effectively rescued these growth defects (Fig 1A). In alignment with its impaired growth, the *sod1* mutant displayed significantly elevated levels of intracellular reactive oxygen species (ROS) during cultivation in YPD (Fig 1B). Collectively, these findings establish that the growth retardation of the *sod1* mutant is primarily driven by endogenous oxidative stress.

**Table 1:** The rates (10^-3^ events per cell division) of genetic alterations in the wild-type (WT) and *sod1*Δ/Δ mutant.

Genome alteration	WT	*sod1*Δ/Δ	*sod1*Δ/Δ (anaerobic)	*sod1*Δ/Δ (5 mM GSH)
SNVs	4.8 (4.5-5.1)	62.5 (56.7-69.1)	4 (2.5-6.1)	27.3 (23.1-32.0)
InDels	0.27 (0.21-0.34)	2 (1.1-3.4)	0.18 (0.005-1)	1.1 (0.4-2.4)
I-LOH	3.3 (3.0-3.5)	50 (44.6-55.7)	3.6 (2.2-5.6)	22.2 (18.4-26.5)
T-LOH	1.3 (1.2-1.5)	21 (17.4-24.6)	1.8 (0.9-3.3)	8.4 (6.1-11.1)
Total-LOH	4.6 (4.3-4.9)	71 (64.4-77.5)	5.5 (3.7-7.8)	30.5 (26.1-35.5)
DEL/DUP	0.18 (0.13-0.24)	1.1 (0.44-2.2)	NA*	NA*
Aneuploidy	0.06 (0.04-0.1)	2.9 (1.8-4.6)	0.18 (0.005-1.0)	0.4 (0.04-1.3)

*NA means no events detected.

The values in the bracket indicate the 95% CI of the rate.

**Fig 1:**
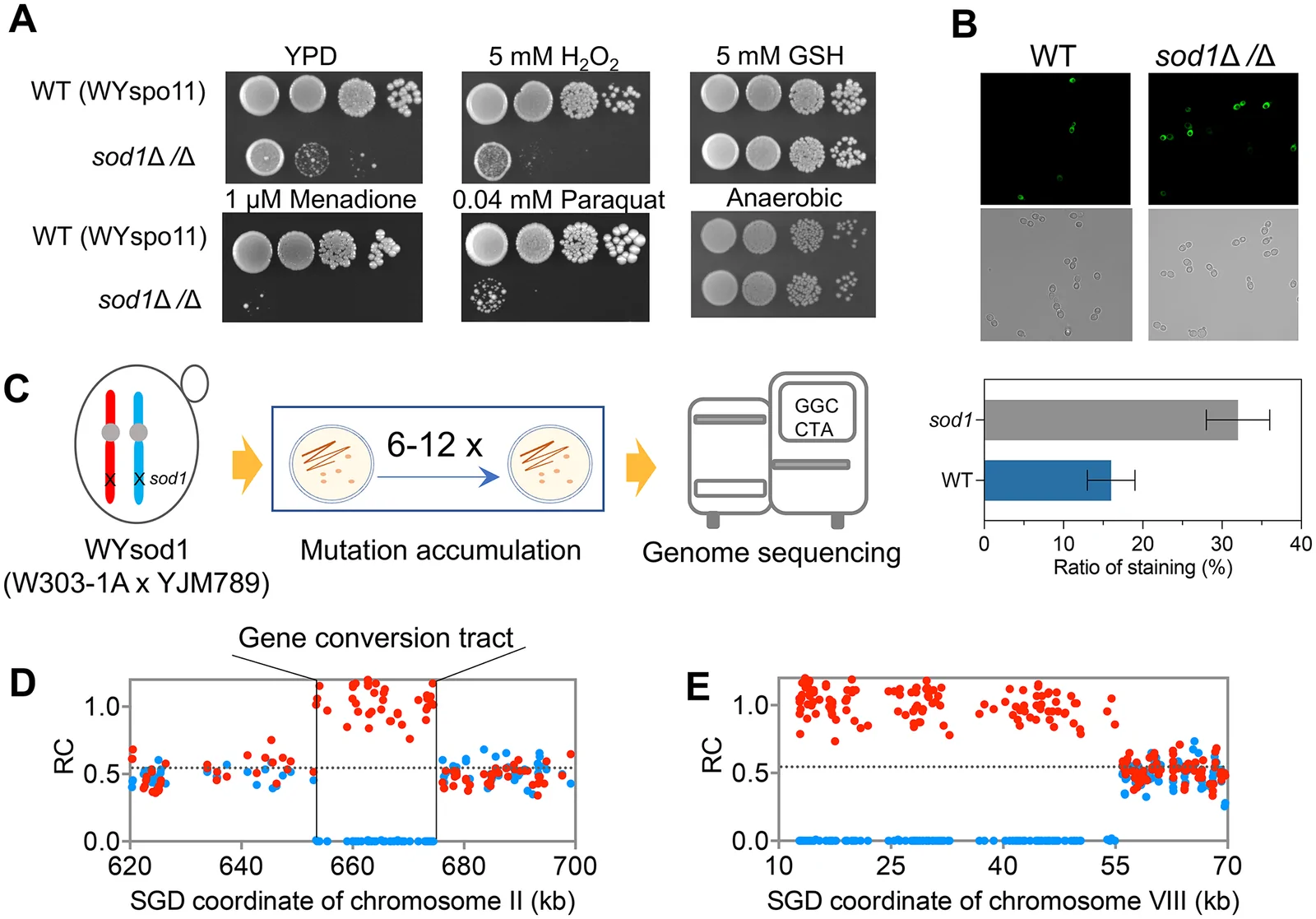
Impact of *sod1Δ*-induced oxidative stress on cellular growth and mitotic recombination. **(A)** Growth comparison between the *sod1Δ/Δ* mutant (WYsod1) and the wild-type strain (WYspo11). Note that the addition of glutathione (GSH) partially rescues the growth defect of the *sod1*Δ/Δ strain. **(B)** Assessment of intracellular ROS production via DCFH-DA staining. A significantly higher proportion of *sod1Δ* cells exhibit positive fluorescence compared to wild-type cells, indicating elevated ROS levels. **(C)** Schematic representation of the Mutation Accumulation (MA) assay using isolates derived from the WYsod1 strain. **(D, E)** Representative whole-genome sequencing (WGS) profiles of *sod1*-induced interstitial LOH (I-LOH, **D**) and terminal LOH (T-LOH, **E**) events. The X-axis represents SGD chromosomal coordinates (kb). The Y-axis shows the ratio of coverage (RC) for allele-specific SNPs from the W303-1A (red) and YJM789 (blue) parental backgrounds. RC values of 0, 0.5, and 1 indicate zero, one, and two copies of the respective homolog, respectively.

To assess genomic stability, 32 *sod1*Δ/Δ isolates were independently subcultured on YPD plates for either 6 (n = 21) or 12 (n = 11) cycles, followed by high-throughput sequencing (Fig 1C). The hybrid genetic background of WYsod1, featuring ~50,000 SNPs between parents W303-1A and YJM789, enabled high-resolution mapping of loss of heterozygosity (LOH) derived from mitotic recombination [19]. Representative LOH events are shown in Fig 1D and 1E; here, red and blue lines denote the relative sequencing coverage (RC) of W303-1A and YJM789 homologs, respectively. For instance, Fig 1D illustrates an interstitial LOH (I-LOH) on chromosome II (652,974–676,158 bp), while Fig 1E shows a terminal LOH (T-LOH) on chromosome VIII (extending from the left telomere to 54,946 bp). In total, we detected 322 I-LOH and 134 T-LOH events across all isolates. The coordinates and patterns of these events are listed in S1 and S2 Datas, respectively. Given an estimated 25 cell divisions per subculture [19], the cumulative divisions totaled 6,450 (21 × 6 × 25 + 11 × 12 × 25). Accordingly, the rates of I-LOH and T-LOH in the *sod1* mutant were 5.0 × 10^-2^ and 2.1 × 10^-2^ events per genome per cell division, respectively—15- and 16-fold higher than those in wild-type cells (Table 1) [19]. Furthermore, the average I-LOH conversion tract length in the *sod1* mutant (10.8 kb, 95% confidence interval [CI]: 7.8–11.3 kb) was longer than that observed in wild-type cells (5.9 kb, 95% CI: 5.3–6.5 kb).

Among the 32 isolates of WYsod1, we identified 7 large deletions/duplications (>1 kb; S3 Data and Fig 2A) and 19 aneuploidy events (Fig 2B). These events occurred at rates of 1.1 × 10^-3^ and 2.9 × 10^-3^ events per genome per cell division, respectively—representing a 4-fold and 48-fold increase compared to wild-type cells (Table 1). Most structural variants were flanked by Ty retrotransposons or LTRs at their breakpoints (S3 Data), suggesting that these rearrangements were driven by homologous sequence-mediated unequal recombination between sister chromatids or non-homologous chromosomes. As illustrated in Fig 2B and S7 Data, aneuploidy in the *sod1* mutant exhibited a strong bias toward trisomy over monosomy, a trend consistent with observations in wild-type cells [19].

**Fig 2:**
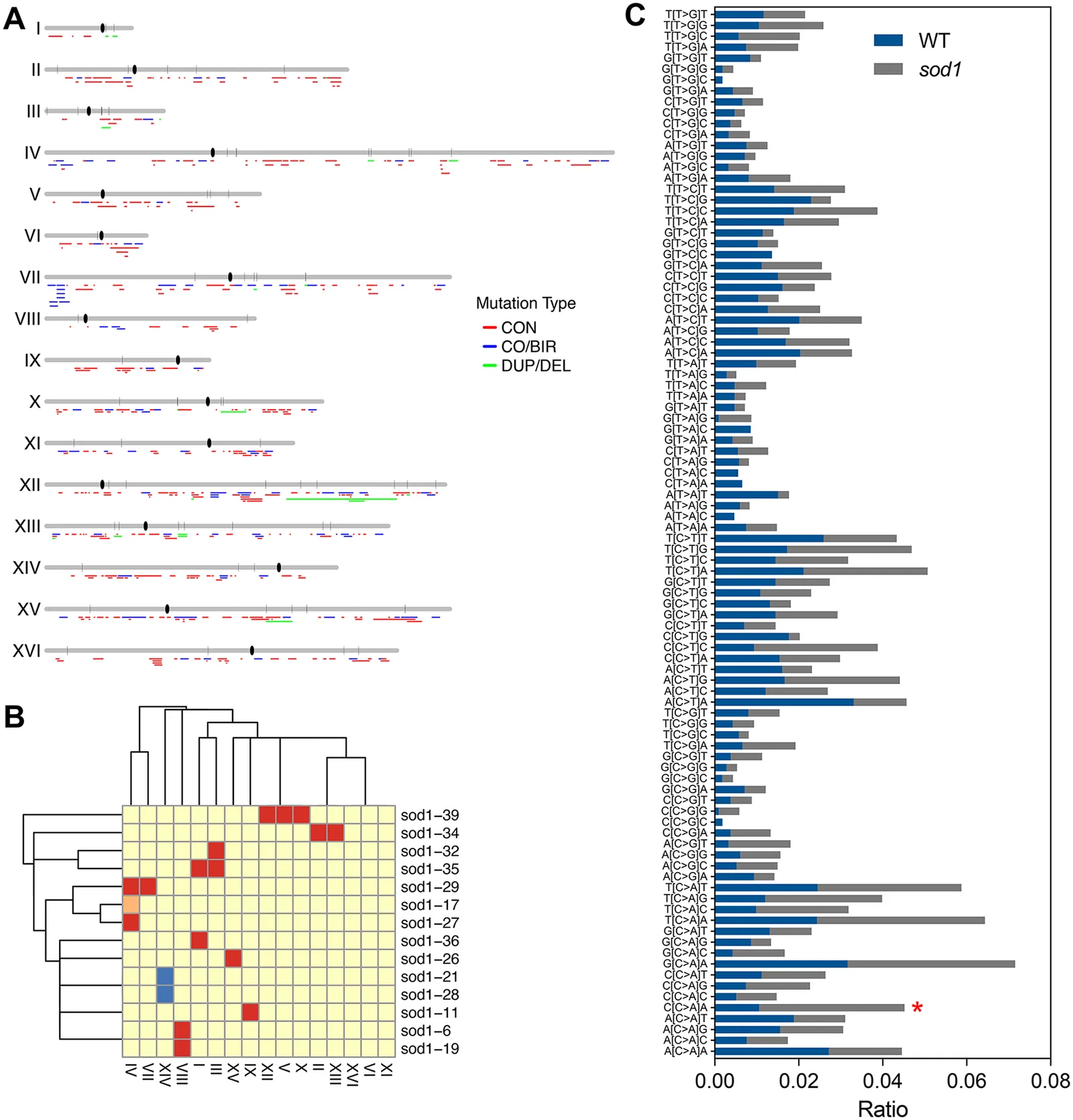
Landscape of mitotic recombination and aneuploidy in *sod1*Δ/Δ mutants. **(A)** Chromosomal distribution of genomic alterations. The map illustrates the genome-wide distribution of interstitial LOH (I-LOH, CON), terminal LOH (T-LOH, CO/BIR), and large-scale deletions/duplications (DEL/DUP) across the 16 yeast chromosomes. For interstitial events (I-LOH and internal DEL/DUP), horizontal bars indicate the precise span of the converted or altered genomic regions. For terminal events, the bars denote the identified breakpoints. **(B)** Profiling of aneuploidy events. A total of 19 aneuploidy events were identified among 14 *sod1*Δ*/*Δ isolates. Color-coded segments represent trisomy (red), monosomy (blue), and uniparental disomy (UPD, orange). **(C)** Analysis of trinucleotide context (the 5’ and 3’ flanking bases) for each type of SNVs. Red asterisk indicates significance at *P* < 0.01 (Chi-square test).

In addition to structural variations, we identified 404 single nucleotide variations (SNVs) and 13 small insertions/deletions (InDels; < 100 bp) within the sequenced *sod1* isolates (S4 Data). The observed rates of SNVs (2.7 × 10^-9^ per bp per division) and InDels (8.8 × 10^-11^ per bp per division) in the *sod1* mutant surpassed wild-type levels by 13-fold and 7.3-fold, respectively (Table 1). To investigate whether SNVs occurred in specific sequence contexts, we analyzed the trinucleotide context (the 5’ and 3’ flanking bases) for each SNV. Notably, our analysis revealed that C-to-A transversions within the 5’-CCA-3’ motif were significantly enriched in *sod1*Δ/Δ cells compared to wild-type cells (Fig 2C). These SNVs and InDels affected 223 genes, resulting in either amino acid substitutions or frameshift mutations (S4 Data). None of the mutated genes were recurrently identified across independent isolates (S4 Data). Nevertheless, Gene Ontology (GO) enrichment analysis revealed that 26.5% of the affected genes were associated with the biological process of cellular response to stimulus, which was significantly enriched compared with the genomic background (P < 0.001, Fisher’s exact test).

To determine whether Sod1 dysfunction induces similar genome instability phenotypes in different genetic backgrounds, we deleted SOD*1* in the haploid strain YJSHM derived from the bioethanol yeast strain YJS329 [20]. The resulting *sod1*Δ mutant YJSHM-sod1 exhibited growth defects under standard rich medium and heightened sensitivity to oxidative stressors (S1A Fig). Using the URA*3* forward mutation assay based on the acquisition of 5-fluoroorotic acid (5-FOA) resistance, we found that SOD*1* deletion increased the URA*3* mutation rate by approximately 5.8-fold in YJSHM cells (S1B Fig).

Collectively, these findings demonstrate that Sod1 deficiency profoundly compromises DNA integrity, triggering elevated global genomic instability in yeast that spans a broad spectrum of genetic lesions—ranging from discrete point mutations to large-scale aneuploidy.

### Anaerobic growth completely diminished genome instability in the *sod1* mutant

To analyze whether the genome instability effect in the *sod1* mutant was originated from oxidative stress, we subcultured 11 *sod1*Δ/Δ isolates on YPD medium for 20 cycles under anaerobic conditions that can rescue the growth defect of *sod1*Δ/Δ cells (Fig 1A). We found that the rate of SNVs in WYsod1 was 1.7 × 10^-10^ per base pair per division, which is comparable to the rate observed in wild-type cells grown under aerobic conditions (Table 1). Similarly, the rate of LOH in the *sod1* mutant decreased significantly under anaerobic conditions (5.5 × 10^-3^ LOH per genome per division) compared to aerobic conditions (Table 1). In addition, we serially passaged 11 independent *sod1*Δ/Δ isolates on YPD medium supplemented with 5 mM GSH for 20 cycles. Whole-genome sequencing revealed that the frequencies of both mutations and LOH events were reduced by more than 50% in *sod1*Δ/Δ isolates cultured in the presence of GSH (Table 1). Thus, our findings indicate that the genomic instability observed in the *sod1*Δ/Δ mutant is primarily due to oxidative stress.

### Approximately 65% of LOH events with an assigned cell-cycle origin in the *sod1*Δ/Δ mutant were initiated by DSBs generated during the G1 phase

According to current genetic models, mitotic recombination in yeast is primarily initiated by double-strand breaks (DSBs) [21–23]. These lesions result in either gene conversion (I-LOH) or crossover events (T-LOH), typically associated with a gene conversion tract proximal to the DSB site (Fig 3A). Reciprocal crossovers originating from DSBs on a sister chromatid during the S/G2 phase exhibit a 3:1 segregation pattern within the conversion tract, whereas those initiated in the G1 phase yield a 4:0 region (Fig 3A). We first confirmed that *sod1* mutants experience a four-fold increase in spontaneous DSB formation compared to wild-type strains, as evidenced by the quantification of Rad52-YFP foci (Fig 3B). To discern the timing of these recombinogenic lesions, we utilized a custom SNP microarray specific to chromosome IV to analyze reciprocal crossovers. The WYsod1 strain is homozygous for the *ade2–1* ochre mutation and carries a single copy of the *SUP4-*o suppressor tRNA gene inserted at the right arm of the YJM789-derived chromosome IV (Fig 3A). Spontaneous crossovers between the centromere and the *SUP4-*o locus produce characteristic white/red sectored colonies (Fig 3A). On YPD medium, the rate of sectored colony formation in WYsod1 was 3.5 ± 1.5 × 10^-4^ per cell division—approximately 10-fold higher than the wild-type rate. This observation aligns with our whole-genome sequencing data, which showed a 14-fold elevation in T-LOH following *SOD1* deletion (Table 1).

**Fig 3:**
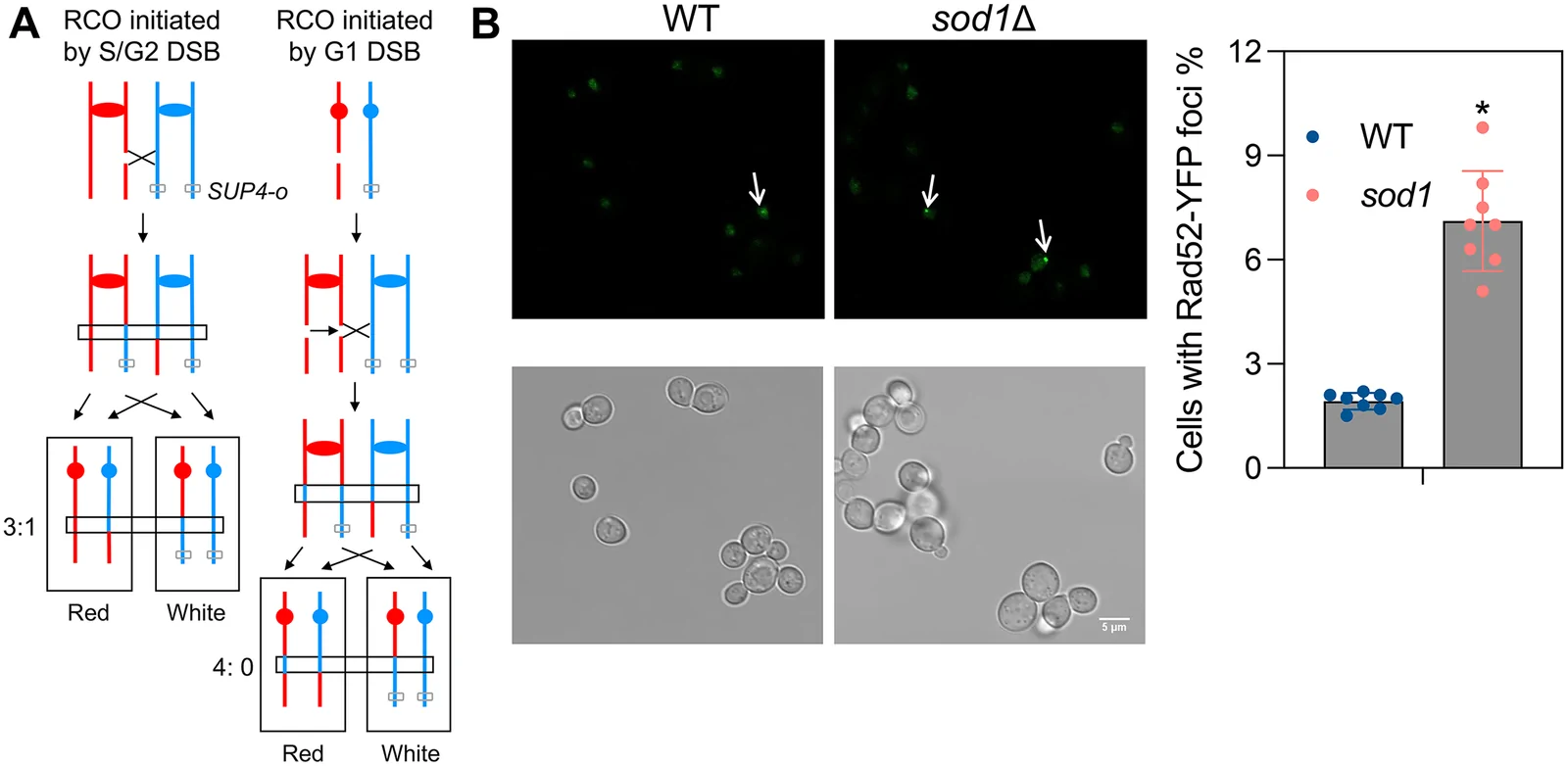
Assessment of recombination timing and DNA double-strand break (DSB) signaling. **(A)** Schematic representation of the colony sectoring assay. The red and blue lines represent chromosomes derived from the W303-1A and YJM789 backgrounds, respectively. This assay distinguishes the cell cycle timing of recombination initiation. Reciprocal crossovers (RCOs) between *CEN4* and the *SUP4-*o marker during the S/G2 phase result in a “3:1” segregation pattern. In contrast, RCOs initiated by DSBs in the G1 phase produce a “4:0” segregation pattern. Daughter cells harboring zero, one, or two copies of the *SUP****4-*o** suppressor gene in an *ade2-1/ade2-1* homozygous background form red, pink, or white colonies, respectively. **(B)** Quantification of Rad52-YFP foci. Representative fluorescence microscopy analysis shows a significantly higher frequency of Rad52-YFP foci in *sod1*Δ mutant compared to the wild-type strain, indicative of elevated spontaneous DNA damage. Data are presented as mean ± SD from multiple independent biological replicates. *P < 0.05 (Student’s t-test).

Representative SNP microarray analysis of a sectored colony is shown in Fig 4A. In the white sector, we identified a YJM789-homologous region extending from 1279 kb to the right telomere of chromosome IV. Conversely, the corresponding region in the red sector was homozygous for W303-1A-derived sequences starting from 1286 kb. This 3:1 segregation pattern within the gene conversion tract indicates that the crossover was triggered by a DSB on the W303-1A-derived chromatid during the S/G2 phase. Among the 35 crossovers characterized in *sod1* isolates (S5 Data), 18 (51%) featured a 4:0 tract, 10 (29%) exhibited a 3:1 pattern, and 7 (20%) had no detectable conversion tract. Collectively, these data suggest that approximately two-thirds (excluding seven events for which the cell-cycle phase could not be assigned) of mitotic crossovers in the *sod1*Δ/Δ mutant are initiated by DSBs occurring in the G1 phase, a mechanistic distribution that closely mirrors the spontaneous recombination patterns observed in WT cells.

**Fig 4:**
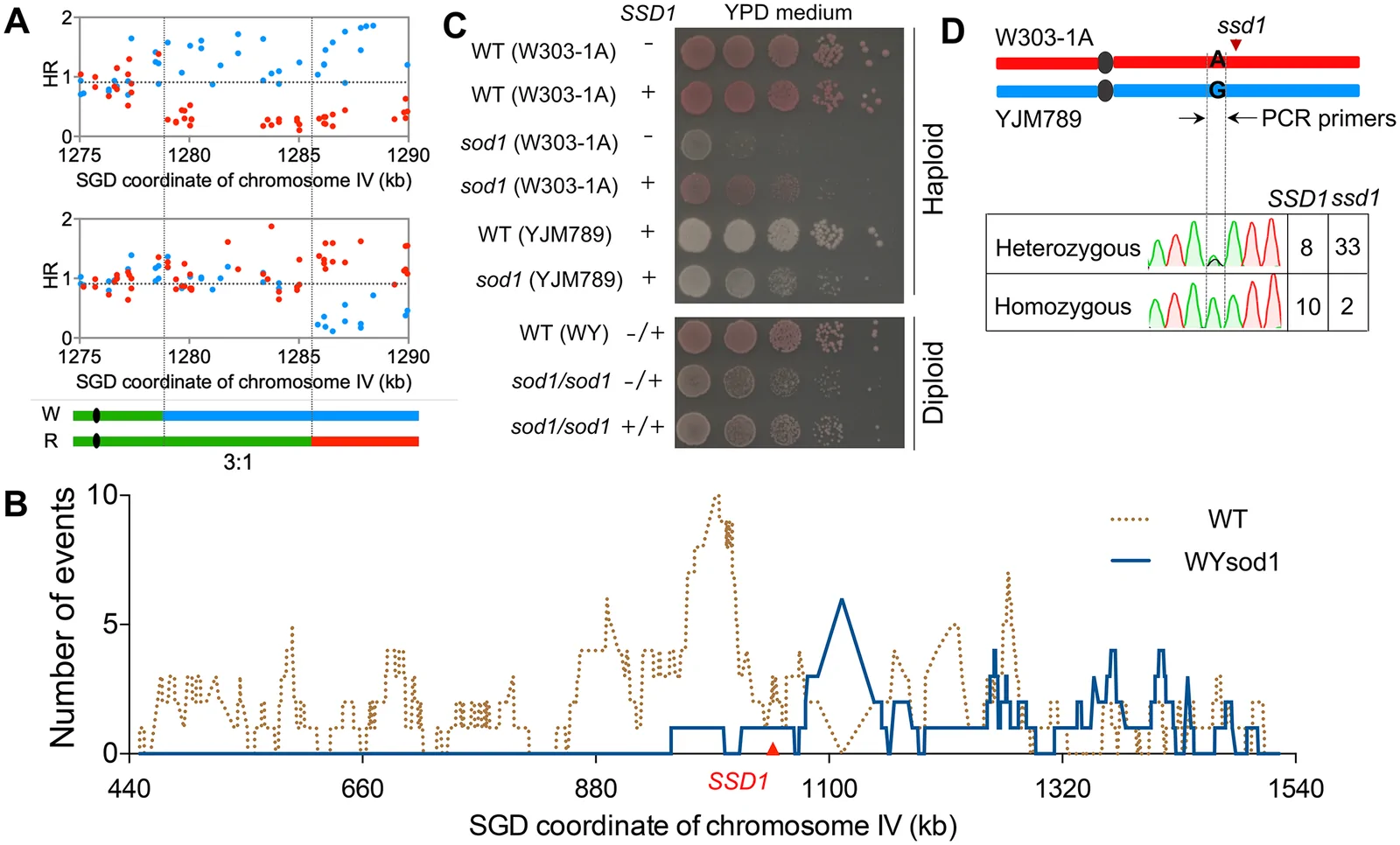
High-resolution mapping of *sod1*Δ-induced crossovers and the modulatory role of the *SSD1* locus. **(A)** Representative SNP microarray profile of a crossover event associated with a “3:1” gene conversion tract. The X-axis represents SGD chromosomal coordinates (kb). The Y-axis shows the normalized hybridization ratio (HR) of W303-1A-derived (red) or YJM789-derived (blue) SNPs in the *sod1Δ* strain relative to a wild-type diploid control. For instance, an HR of ~1.5 for YJM789 SNPs (blue) and ~0 for W303-1A SNPs (red) at the distal end of chromosome IV indicates that the white (W) daughter colony inherited two copies of the YJM789 homolog and zero copies of the W303 homolog from the breakpoint (1279 kb) to the telomere, as depicted in the schematic below. Green bars denote hybrid (recombinant) chromosomes. **(B)** Genomic distribution of crossover breakpoints along chromosome **IV.** Crossover frequencies are compared between the wild-type strain (brown line) and the *sod1*Δ strain (blue line). The *SSD1* locus (1049 kb) is indicated by a red triangle. **(C)** Phenotypic analysis of the genetic interaction between *SSD1* and *SOD1*. Serial dilution spot assays were performed on YPD plates to assess the fitness of parental haploids and diploids with the indicated *SOD1* and *SSD1* genotypes. **(D)** Analysis of breakpoint clustering relative to the *SSD1* locus by PCR. Detection of heterozygosity at the SNP site nearest to *SSD1* revealed that upon restoration of the functional *SSD1* allele, crossover breakpoints were distributed uniformly around the *SSD1* locus, in contrast to their clustering to the right of the *ssd1* mutation in the original mutant background.

### LOH breakpoints were non-randomly distributed on chromosomes in *sod1* mutant

Notably, 33 of the 35 crossover events identified on the right arm of chromosome IV exhibited breakpoints clustered between 1050 kb and the distal telomere (Fig 4B). We hypothesized that this biased distribution arises from fitness costs; specifically, crossover-induced LOH near the 1050 kb locus might cause significant viability differences between daughter cells, thereby precluding the formation of visible sectored colonies. The W303-1A background harbors a nonsense mutation in *SSD1* (coordinates 1,045,640–1,049,392 bp), a gene critical for cell wall stability and cell cycle progression [24,25]. To determine if Ssd1 deficiency drives this breakpoint bias, we restored the wild-type *SSD1* allele in both W303-1A and the derived *sod1* mutant. While wild-type growth remained unchanged, *SSD1* restoration significantly improved the fitness of the *sod1*Δ/Δ mutant (Fig 4C).

In the sectored colony assay, the *sod1* mutant homozygous for wild-type *SSD1* showed a crossover rate (4.7 ± 1.1 × 10^-4^ per division) comparable to that of WYsod1. Crucially, however, breakpoints were no longer restricted to the 1050 kb-telomere region, as confirmed by PCR and Sanger sequencing of the SNP at SGD coordinate 1,045,331 bp (Fig 4D). Furthermore, *sod1*Δ cells carrying two functional *SSD1* copies exhibited a distinct growth advantage over those with only one copy (Fig 2D), indicating *SSD1* haploinsufficiency in a *sod1*Δ/Δ background. Collectively, these findings demonstrate that Ssd1 and Sod1 deficiencies synergistically impair cellular fitness, leading to a recovery bias in observed crossover events.

We further analyzed whether specific chromosomal features—such as Ty elements, LTRs, G4 motifs, or replication termination sites—correlated with LOH breakpoints. Enrichment analysis showed no significant association with these elements (S6 Data). Interestingly, however, we found that regions with low GC content (<36%) were significantly underrepresented near LOH breakpoints in sod1 mutants.

### C:G to A:T is the most prominent base substitutions in the *sod1* mutant

While all six base substitution types significantly increased in the *sod1* mutant (7- to 18-fold relative to wild-type), transversions emerged as the predominant signature (Fig 5A). Consequently, the proportion of these transversions was significantly elevated in *sod1*Δ/Δ cells compared to wild-type controls (P < 0.05, Fisher’s exact test; Fig 5B). Guanine is known to be highly susceptible to oxidation, particularly through the addition of hydroxyl radicals to the C8 position to form 8-oxo-7,8-dihydroxy-2′-deoxyguanosine (8-oxo-dG) [26,27]. Given that most DNA polymerases preferentially insert dA opposite 8-oxo-dG [28], this lesion typically results in G to T transversions after DNA replication.

**Fig 5:**
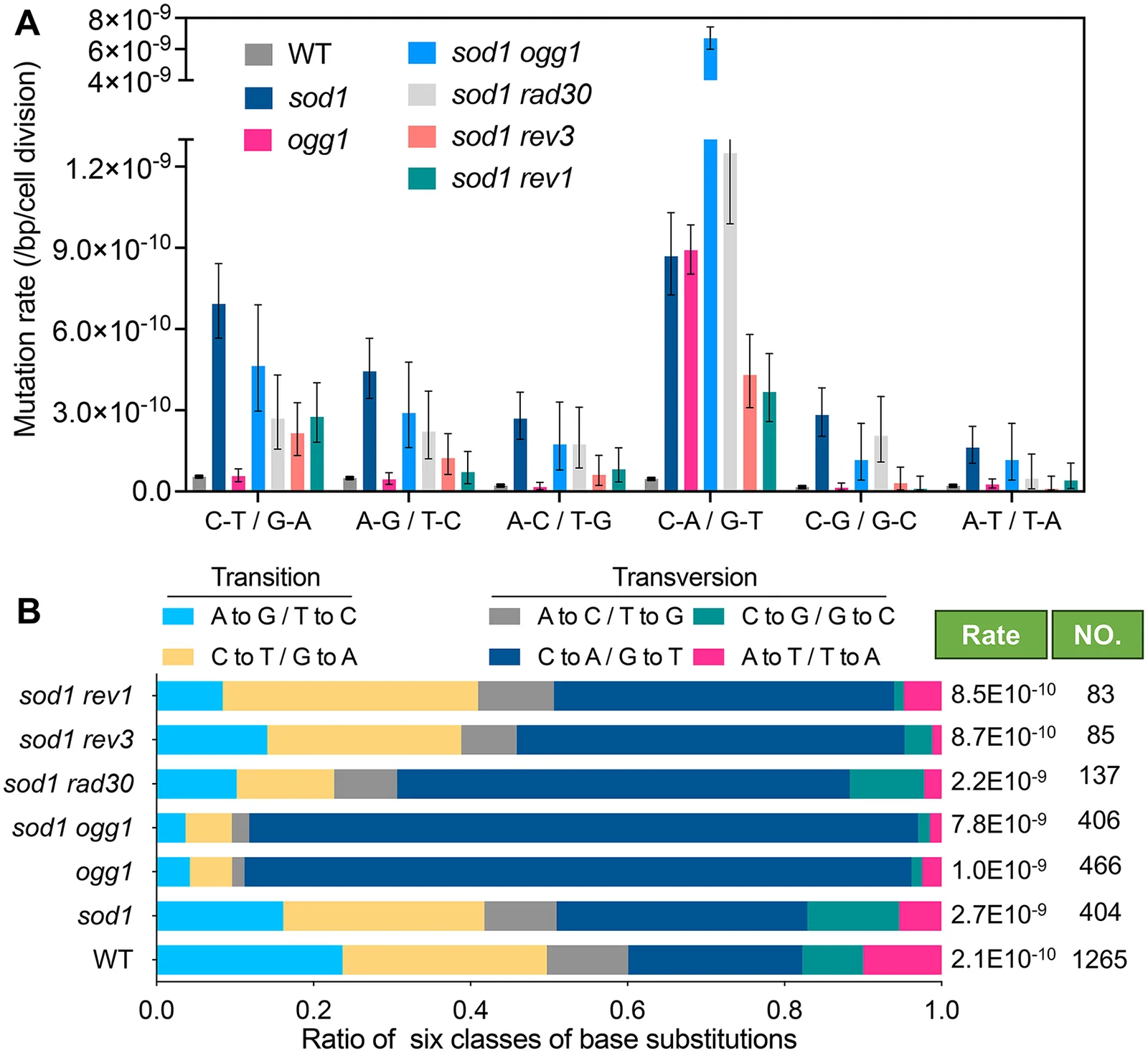
Contribution of the base excision repair (BER) and translesion synthesis (TLS) pathways to *sod1*Δ-induced point mutations. **(A)** Specific rates of single-base substitutions. The graph depicts the rates of individual substitution types (per bp per division) across isogenic diploid strains: wild-type (WYspo11), *ogg1*Δ (SY176), *sod1*Δ (WYsod1), and the double mutants *sod1*Δ *ogg1*Δ (SY146), *sod1*Δ *rad30*Δ (SY268), *sod1*Δ *rev3*Δ (SY269), and *sod1*Δ *rev1*Δ (SY270). These data illustrate the genetic dependencies of oxidative DNA damage repair and bypass. **(B)** Total SNV rates and mutational spectra. The cumulative SNV rates (right) and the distribution of the six classes of nucleotide substitutions (left) are shown for the indicated genotypes.

To test whether 8-oxo-dG accumulation drives the observed mutational bias, we deleted *OGG1* in both wild-type and *sod1* backgrounds. *OGG1* deletion alone triggered a 19-fold increase in C:G to A:T transversions without affecting other substitution types (Fig 5A), InDels, or LOH, confirming that Ogg1-mediated base excision repair (BER) is the primary *in vivo* defense against 8-oxo-G. Notably, the *sod1 ogg1* double mutant exhibited a 7.5-fold higher C:G to A:T transversion rate compared to the *ogg1* single mutant, and a 9.6-fold increase compared to the *sod1* single mutant (Fig 5A). These data indicate that the Ogg1-mediated BER pathway effectively repairs approximately 90% of the 8-oxo-G:dA mismatches generated in the absence of Sod1 (calculated as (9.6 - 1)/9.6 = 90%).

### DNA polymerase (pol) η decreased C:G to A:T substitutions in *sod1* cells

Previous studies established that DNA polymerase η (Pol η, encoded by *RAD30*) preferentially inserts cytosine (C) opposite 8-oxo-dG during replication [28], a mechanism postulated to suppress C:G to A:T transversions in *sod1*Δ/Δ mutant. To elucidate the role of Pol η in oxidative stress-induced mutagenesis, we deleted *RAD30* in both diploid wild-type and *sod1* strains and performed a 15-generation subculturing experiment. Whole-genome sequencing of eight isolates revealed that the overall SNVs rate in the *sod1 rad30* double mutant (2.2 × 10^-9^ SNVs per base per division) was 18% lower than that of the *sod1* single mutant (Fig 5B). Conversely, the rate of C:G to A:T transversions (1.3 × 10^-11^ SNVs per base per division) exhibited a 1.5-fold increase (Fig 5A), accounting for 58% of all base substitutions (Fig 5B). These data indicate that Pol η prevents approximately 34% of C:G to A:T mutations in the absence of Sod1. Collectively, our findings demonstrate that while Pol η acts as an error-prone polymerase contributing to the general mutation burden under oxidative stress, it specifically mitigates the occurrence of C:G to A:T transversions.

### DNA Pol ζ contributed to the mutagenesis of Sod1 deficiency

DNA polymerase ζ (Pol ζ), with Rev3 as its catalytic subunit, is a key mediator of translesion synthesis (TLS) in eukaryotes [29–31]. As shown in Fig 5B, the SNVs rate in the *sod1 rev3* double mutant was only 31% of that observed in the *sod1* single mutant, indicating that approximately two-thirds of *sod1*-induced SNVs are Pol ζ-dependent. However, *REV3* deletion exerted distinct effects on specific base substitution types. Notably, the rates of G:C to C:G and A:T to T:A substitutions in the *sod1 rev3* mutant were reduced to 11% and 6% of *sod1* levels (Fig 5B), respectively, demonstrating a near-absolute requirement for Pol ζ. In contrast, the C:G to A:T transversion rate decreased by only 52% in the absence of Rev3 (Fig 5B), suggesting this specific mutation type is comparatively less dependent on Pol ζ activity. Consequently, the relative proportion of G:T transversions shifted from 32% in the *sod1* single mutant to 49% in the *sod1 rev3* double mutant (Fig 5B).

The *REV1* gene encodes a deoxycytidyl transferase that preferentially incorporates C opposite abasic sites and interacts with Pol ζ to facilitate lesion bypass and extension [32,33]. Deletion of *REV1* reduced the overall SNVs rate in the *sod1* mutant by 67%, mirroring the 69% reduction seen in *rev3 sod1* strain (Fig 5B). Consistent with the result of *sod1 rev3* double mutant, G:C to C:G and A:T to T:A substitutions were drastically diminished (>80%) in the *sod1 rev1* double mutant (Fig 5B). Collectively, these results demonstrate that Rev1 and Pol ζ function within the same pathway to bypass oxidative base damage, accounting for the majority of SNVs in *sod1*-deficient cells.

### Certain ALS-associated mutations of Sod1 compromise genome integrity

Approximately 20% of fALS cases are linked to mutations in the *SOD1* gene, including variants such as A4V, G36R, H48Q, G85R, N86S, D90A, G93A, and S133N [33,34]. To investigate whether these ALS-associated substitutions impair genomic stability, we expressed wild-type human *SOD1* and eight clinical variants from *CEN/ARS* plasmids in the *sod1*Δ/Δ strain (WYSod1; Fig 6A). Following 10 subculturing cycles, whole-genome sequencing of six isolates per variant revealed that the H48Q, G85R, N86S, and S133N mutants exhibited SNVs rates ranging from 2.3 × 10^-9^ to 2.8 × 10^-9^ per bp per division (Fig 6B). These rates are comparable to those observed in the *sod1*Δ/Δ mutant (Table 1). Furthermore, LOH frequencies were elevated in these four specific mutants relative to the strain expressing wild-type human *SOD1* (Fig 6B). In contrast, the A4V, G36R, D90A, and G93A variants did not significantly alter the rates of these genetic events (Fig 6B).

**Fig 6:**
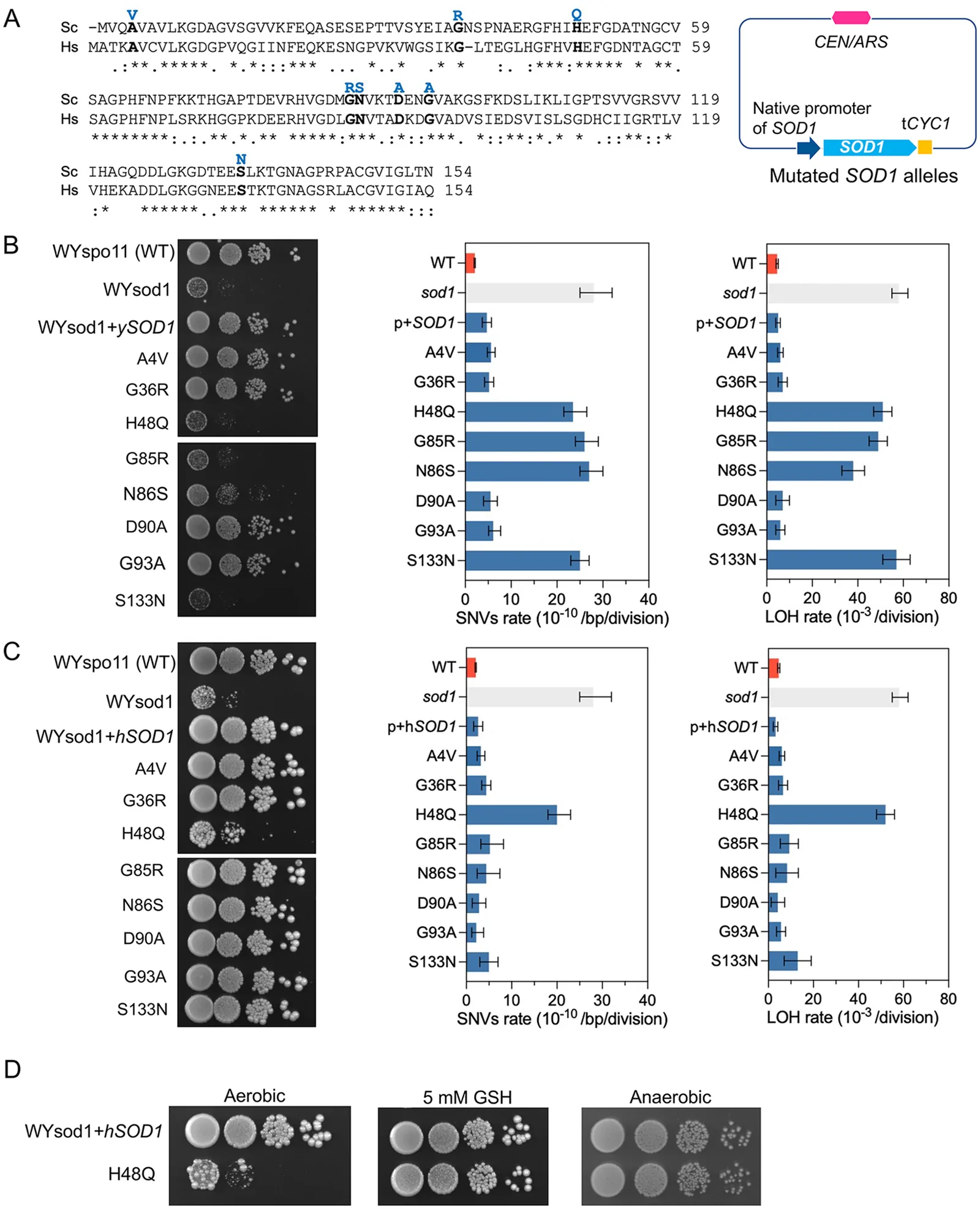
Comparative analysis of genomic stability across Sod1 isoforms and ALS-associated variants. **(A)** Comparison of yeast and human Sod1 protein sequences and common amino acid substitutions associated with ALS (left panel). Heterologous expression system for *SOD1* isoforms (right panel). Schematic representation of the *CEN/ARS* plasmid-based expression of various Sod1 amino acid substitution mutants in the *sod1*Δ/Δ strain (WYsod1). Expression of the *SOD1* variants was driven by the endogenous yeast *SOD1* promoter. **(B, C)** Functional impacts of yeast and human Sod1 isoforms on cellular fitness and genome integrity. Assessment of cell growth (left) and the rates of SNVs and LOH per cell division (right). Data for yeast-derived isoforms are presented in **(B)**, while human-derived isoforms are shown in **(C)**. Error bars denote 95% confidence intervals (CIs) estimated by a Poisson distribution. **(D)** Growth comparison of wild-type human *SOD1*- and H48Q-expressing strains under aerobic, GSH-supplemented, and anaerobic conditions. **(E)** In the YJSHM background, the H48Q mutant exhibited significantly reduced mutation rates under anaerobic conditions and upon GSH (5 mM) supplementation, as determined by the forward mutation assay using the report gene *URA3*. Data are presented as mean ± SD from three independent biological replicates. *P < 0.05 (Student’s t-test).

Expression of wild-type human *SOD1* effectively rescued the growth defect of yeast cells lacking endogenous *SOD1* (Fig 6C), demonstrating high functional homology. This complementation allows for the precise characterization of human *SOD1* point mutations within a yeast model. Notably, while the H48Q mutation significantly inhibited cell growth, the other variants showed no obvious growth phenotypes (Fig 6C). From a genomic perspective, the H48Q substitution induced an 8- and 10-fold increase in SNVs and LOH frequencies, respectively, whereas the G85R and S133N mutations exerted only moderate effects (less than 3-fold increase) (Fig 6C). Similar to complete loss of *SOD1*, both anaerobic conditions and GSH supplementation alleviated the growth defect (Fig 6D). These results suggest that specific ALS-linked *SOD1* mutations may contribute to disease pathogenesis by disrupting redox homeostasis and compromising genome integrity.

## Discussion

In this study, we employed a yeast model to systematically elucidate the impact of superoxide dismutase (Sod1) deficiency on genomic integrity. Our findings demonstrate that Sod1 serves as a critical guardian of the genome, where its absence triggers a cascade of instability ranging from single-nucleotide variations to large-scale chromosomal rearrangements at the whole genome level.

### The primary driver and patterns of genomic instability in *sod1* mutant

While Sod1 has been previously characterized as a transcription factor that modulates the expression of DNA repair genes [13], our findings suggest that the predominant driver of genomic instability in *sod1*Δ/Δ strain is the direct chemical impact of endogenous ROS. The complete rescue of both mutation rates and LOH frequencies under anaerobic conditions provides compelling evidence that oxygen-derived radicals are the primary mutagenic agents (Table 1). Furthermore, our analysis of the *sod1 ogg1* double mutant reveals a synergistic increase in the characteristic C:G to A:T transversion frequency compared to the *ogg1*Δ single mutant (Fig 5). This observation is critical: if ROS were primarily inactivating DNA repair enzymes or replication proteins, the additional loss of Sod1 in an *ogg1*Δ background (where the repair of 8-oxoguanine is already compromised) would not be expected to so dramatically exacerbate the specific mutational signature. Therefore, our results underscore that ROS promote mutagenesis primarily by directly inducing DNA lesions (such as base oxidation and DNA breaks) rather than through the systemic oxidative inactivation of the proteome involved in genome maintenance.

Previous studies indicate that in wild-type strains, approximately 75% of spontaneous mitotic recombination events are likely initiated by DSBs occurring in the G1 phase [35]. In *sod1*Δ/Δ mutants, this proportion slightly decreased to 65%, suggesting that ROS-induced oxidative stress directly contributes to DSB formation during G1. However, it is essential to highlight that DSBs occurring in the S/G2 phase are likely more frequent than those in G1 in both wild-type and *sod1*Δ/Δ cells. Because S/G2 DSBs are preferentially repaired using the sister chromatid as a template—a process that does not result in LOH—these events remain largely undetected in our experimental system [35,36]. Given that ROS are particularly prone to damaging single-stranded DNA (ssDNA), for instance, the •OH can directly abstract a hydrogen atom from the deoxyribose sugar ring [37]. This reaction generates a carbon-centered radical on the sugar moiety, leading to sugar ring fragmentation and cleavage of the phosphodiester backbone, thereby producing a single-strand break (SSB) [37]. In *sod1*Δ/Δ cells, accumulation of oxidative ssDNA lesions during DNA replication may cause replication fork collapse, generating replication-associated DSBs that drive the high frequency of LOH events observed in these cells. In addition, closely spaced SSBs on opposite DNA strands can be converted into DSBs independently of DNA replication [16].

Beyond the increase in event frequency, we observed that the gene conversion tracts associated with I-LOH were significantly longer in *sod1*Δ/Δ mutants compared to wild-type cells. The initiation of homologous recombination requires 5’-3’ resection of the DSB ends to expose 3’ single-stranded DNA overhangs (Fig 7). We speculate that two possible mechanisms may contribute to the expanded gene conversion tracts observed in *sod1*Δ/Δ cells. First, the oxidative environment resulting from Sod1 deficiency may enhance the activity of 5’-3’ exonucleases, leading to more extensive resection. Alternatively, the exposed 3’ overhangs might be directly targeted and broken by ROS. Such damage would necessitate additional rounds of 5’-3’ resection to generate a new, sufficiently long 3’ end capable of invading the homologous template, ultimately manifesting as elongated gene conversion tracts in the resulting recombinants.

**Fig 7:**
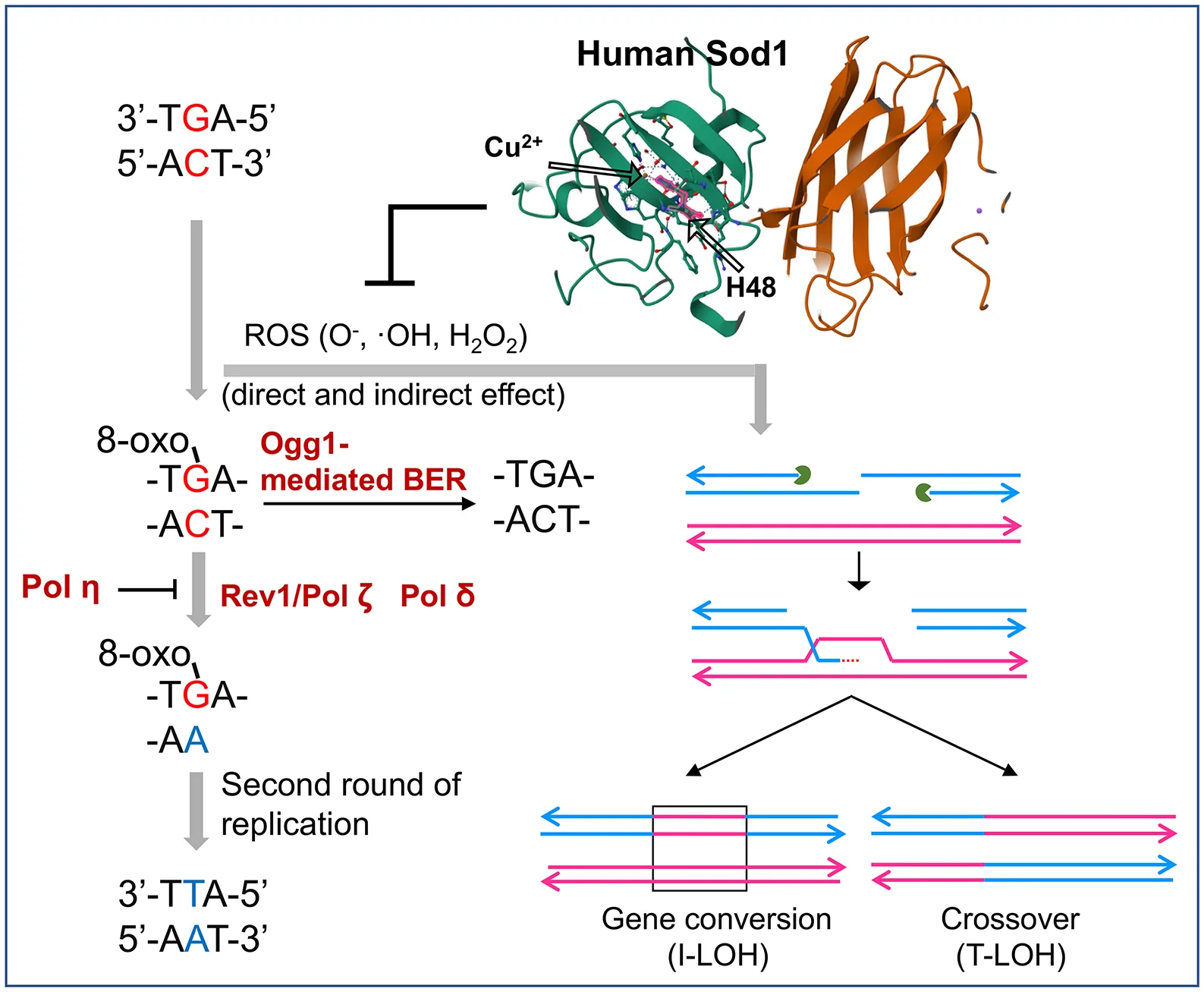
Proposed mechanisms of genomic instability induced by Sod1 deficiency. Sod1 acts as a critical antioxidant shield by neutralizing reactive oxygen species. In contrast, the H48Q mutant loses its metal-binding ability and subsequent enzymatic function. Unscavenged ROS leads to the formation of 8-oxoG lesions. These lesions are primarily repaired by glycosylase Ogg1-mediated BER pathway. In the absence of efficient repair, TLS polymerases bypass the 8-oxoG site: DNA polymerase η facilitates error-free bypass, while the Rev1/Pol ζ complex promotes error-prone bypass, resulting in the characteristic C:G to A:T transversions observed after a second round of replication. Elevated ROS levels directly or indirectly trigger DNA breaks and elevated rates of DNA recombination and LOH.

The genome-wide mapping of LOH breakpoints in *sod1*Δ/Δ mutants revealed a lack of prominent, pre-defined recombination hotspots (Fig 2A), suggesting that oxidative stress-induced DNA damage is relatively stochastic in its distribution. However, the frequency of these events is modulated by local sequence characteristics. Our correlation analysis indicated that regions with low GC content exhibit a significantly reduced frequency of recombination. Given that guanine is the most easily oxidized base, it is plausible that low-GC regions are inherently more resistant to the formation of 8-oxoguanine and subsequent strand breaks. Interestingly, while low GC content appears protective, high GC content alone was not sufficient to create enrichment hotspots for breakpoints, suggesting that other factors—such as chromatin accessibility or transcriptional activity—may also contribute to the local susceptibility to ROS.

Our results further highlight the critical role of post-mutational selection in shaping the observed genomic landscape. A striking example is the biased distribution of T-LOH events on chromosome IV (Fig 4B). The synergistic growth defect caused by *ssd1* and *sod1* mutations imposes a strong fitness disadvantage on cells that lose the functional *SSD1* allele during recombination. Therefore, the enrichment of specific T-LOH events on chromosome IV in the WYsod1 strain likely reflects selective retention of fitter recombinants rather than an intrinsic bias in DSB formation or recombination initiation. Consistent with this notion, the distribution of functional mutations was also non-random. Annotation of SNVs and InDels that caused amino acid substitutions or frameshift mutations revealed that the affected genes were significantly enriched in specific functional categories (S4 Data). GO enrichment analysis suggested that genes involved in cellular response to stimulus were preferentially targeted or retained among the evolved isolates. One possibility is that mutations in these genes provide a selective advantage by improving adaptation to the chronic oxidative stress associated with *SOD1* deficiency. Alternatively, these genes may be less constrained for essential cellular processes, allowing functional alterations to be tolerated without severely compromising growth. In addition to sequence-level mutations, chromosome-scale alterations also contributed to genome evolution in *sod1*Δ/Δ isolates. We observed that trisomic chromosome events were more frequent than monosomic events, with chromosome I trisomy independently occurring in two isolates (S7 Data). Previous studies have demonstrated that aneuploidy can serve as a rapid adaptive strategy in yeast by altering gene dosage and promoting stress tolerance. Thus, chromosomal abnormalities observed in *sod1*Δ/Δ isolates, including chromosome I trisomy, may partially compensate for the fitness defects caused by Sod1 loss, although the specific adaptive advantages conferred by these alterations require further investigation. Together, these findings suggest that the genomic landscape generated by oxidative stress is shaped by a two-step evolutionary process: the initial generation of DNA lesions and genome alterations determined by intrinsic molecular vulnerability (e.g., sequence context such as GC composition), followed by selection that filters and enriches variants with improved cellular fitness.

### The mutational landscape and the underlying genetic mechanisms

A hallmark of Sod1 deficiency is the enrichment of C:G to A:T transversions that preferably occurred in 5’-CCA-3’ (Fig 2C). This signature is a direct consequence of 8-oxo-G accumulation, which induces mispairing with adenine during replication (Fig 7). Our data highlight a sophisticated hierarchy of DNA repair and bypass mechanisms. The BER glycosylase Ogg1 serves as the frontline defense, correcting the vast majority of 8-oxo-G lesions. When BER is overwhelmed or absent, translesion synthesis polymerases dictate the mutational outcome (Fig 7). We found that Pol η (Rad30) acts protectively by facilitating relatively accurate bypass (could be explained by Pol η replicate DNA containing 8-oxo-G efficiently and accurately by inserting a cytosine across from the lesion), while Pol ζ (Rev3) and Rev1 are responsible for the majority of the remaining transversions (Fig 7). This error-prone bypass ensures the completion of DNA replication at the cost of increased mutational load, illustrating a critical trade-off in cells under high oxidative stress.

The dual role of ROS in cancer biology presents a therapeutic window that our study may help refine. Cancer cells frequently exhibit aberrant redox homeostasis; while moderate ROS levels promote pro-tumorigenic signaling and hyperproliferation, excessive ROS production is cytotoxic [38]. To survive this high oxidative burden, tumor cells often adapt by upregulating antioxidant defenses, including Sod1, to maintain a redox balance that optimizes growth [39,40]. This dependency has led to the development of Sod1 inhibitors, such as ATN-224, as potential anti-cancer agents [41]. However, our work sounds a cautionary note regarding such strategies. By demonstrating that Sod1 deficiency leads to a global surge in genomic instability—including point mutations, chromosomal rearrangements, and aneuploidy—we suggest that the pharmacological inhibition of Sod1 may inadvertently accelerate the genomic evolution of tumors. While these drugs may achieve initial cytotoxicity, the resulting “mutational burst” could facilitate the rapid emergence of diverse DNA variations and the subsequent acquisition of drug-resistant phenotypes. Future clinical applications of Sod1 inhibitors may therefore require co-treatment strategies to suppress the inevitable increase in genomic erosion and compensatory evolution.

### Genetic effects of ALS-associated Sod1 variants in yeast model

Our results demonstrate that wild-type human Sod1 can effectively rescue the growth defects and genomic instability of yeast *sod1*Δ/Δ strains (Fig 6C), reinforcing the high degree of functional conservation between these two species. This cross-species complementation validates the yeast system as a robust “living test tube” for characterizing the functional consequences of human clinical variants. Given the rapid turnover and well-defined genetic background of yeast, it serves as a sensitized model capable of detecting subtle physiological impairments that might be technically challenging to monitor in long-lived human motor neurons.

A significant finding in this study is the striking heterogeneity among the eight ALS-associated Sod1 variants tested. While the “toxic gain-of-function” through protein misfolding and aggregation is the prevailing hypothesis for Sod1-linked ALS [42,43], our data suggest that loss of genomic protection may be an additional, mutation-specific pathogenic driver. Specifically, the H48Q and S133N variants significantly compromised genome integrity, with H48Q exhibiting a near-null phenotype in terms of both growth and mutation rates. Notably, the H48 residue is critical for copper-binding for both human and yeast Sod1 based on structure analysis (Fig 7) [44,45]; its mutation likely disrupts the enzyme’s catalytic core, leading to a surge in endogenous ROS and subsequent DNA damage. This indicates that for a subset of ALS patients carrying genotoxic variants like H48Q, the primary insult may be a failure to quench nuclear ROS, leading to a progressive loss of genomic information that precedes or exacerbates protein-level toxicity. In contrast, variants like A4V and G36R did not significantly elevate mutation rates in our model, suggesting that their pathogenicity may stem from different mechanisms, such as increased propensity for aggregation rather than the loss of antioxidant-mediated genome surveillance.

The observed discrepancy in mutational impact between human and yeast Sod1 may be attributed to a fundamental divergence in evolutionary strategies for protein robustness. Human Sod1 is characterized by extraordinary thermal stability and structural redundancy; the mature hSod1 dimer remains remarkably stable even in its metal-deficient states [46,47]. This high level of intrinsic stability likely reflects an evolutionary adaptation in long-lived species to provide mutational tolerance, thereby safeguarding the proteome against the cumulative burden of somatic mutations over several decades. In contrast, the single cell organism *S. cerevisiae*, with its significantly shorter life cycle, may have evolved a Sod1 variant optimized for rapid catalytic turnover or metabolic efficiency at the expense of extreme structural resilience. We hypothesize that specific amino acid substitutions, which might only marginally perturb the conformational energy landscape of the robust human Sod1, could exceed the proteostatic threshold of the more fragile yeast Sod1. Such a collapse in folding kinetics would lead to a profound loss of antioxidant capacity, ultimately manifesting as the severe genomic instability phenotypes observed in the yeast model.

In summary, our work provides a comprehensive map of the genomic consequences of Sod1 loss in yeast model. By defining the mutational signatures, repair dependencies, and chromosomal vulnerabilities associated with oxidative stress, we establish a framework for understanding how antioxidant deficiencies contribute to genome instability. Our results also indicate the loss of genomic protection as a genotoxic driver in the pathogenesis of specific ALS-linked *SOD1* variants.

## Materials and methods

### Ethics statement

This article does not contain any studies with human participants or animals performed by any of the authors.

## Strain construction and medium

All diploid strains used in this study were generated by crossing haploid strains with W303-1A and YJM789 backgrounds. Yeast transformation was performed using the classical LiAc/ssDNA/PEG method [48]. The detailed protocols for strain construction are described in S1 Text (Supplemental file). YPD medium containing 20 g/L glucose, 20 g/L peptone, 10 g/L yeast extract was used to culture yeast. To maintain the genomic stability, 2.5 mM GSH was added on the YPD plates before subculturing of the *sod1*Δ/Δ mutant strains.

### Intracellular ROS determination

Log-phase yeast cells (∼3  ×  10^6^) were harvested and resuspended in 1 mL of 1 × phosphate-buffered saline (PBS) (10 mM phosphate, 138 mM NaCl, and 2.7 mM KCl, pH 7.4). DCFH-DA was added to a final concentration of 10 μM, and the cells were incubated at 30°C for 1 h. After washing three times with 1 × PBS, the fluorescence of each sample was measured using a multimode microplate reader (Synergy H1; Biotek) at the longest excitation wavelength (488 nm) and the maximum emission wavelength (525 nm). Raw data were collected and the mean values of five replicates was used for statistical analysis.

### SNP microarray analysis

Chromosome IV specific SNP microarrays were used to detect the genetic events in the sectored colonies of *sod1*Δ/Δ strain. The protocols of genome preparation, hybridization of sample and control DNA, and data analyses were described in previous studies [49,50].

### Whole genome sequencing and data analysis

Genomic DNA was extracted from yeast isolates using the Omega Yeast DNA Kit (Life Science Products). Whole-genome sequencing was performed using a 150-bp paired-end strategy on either the Illumina NovaSeq or MGISQ 2000 platforms. Genomic alterations were identified as previously described [51,52]. Briefly, raw sequencing reads were aligned to the *S. cerevisiae* S288c reference genome using BWA to obtain .sam files [53]. Samtools was used to convert the .sam files to .mp files [54], followed by SNVs and InDels calling using the software VarScan 2.3.9 [55].

LOH was defined as an event involving the simultaneous duplication of one allele and the deletion of the corresponding homologous allele at one or more heterozygous SNP loci. Chromosomal deletions or duplications were determined based on the ratio of coverage (RC), defined as the read depth at a specific heterozygous SNP normalized to the genome-wide average depth. A deletion was called when the RC was below 0.05, while a duplication was defined by an RC exceeding 0.7.

### *URA3* forward mutation assay

*URA3* mutation rates were determined by measuring the frequency of 5-FOA–resistant (5-FOA^r^) mutants across 20 independent cultures. The resulting frequency data were converted to mutation rates as described by Kiktev et al. [56]. The Ura- phenotype of these 5-FOA^r^ isolates was confirmed by replica-plating onto uracil-deficient synthetic complete (SC-Ura) medium.

### Sod1 isoforms construction

The wild-type *S. cerevisiae SOD1* allele, including its ORF and 500 bp of the upstream promoter sequence, was amplified from W303-1A genomic DNA. The resulting fragment was inserted into the *CEN/ARS* vector pYXZ1 [57] via Gibson assembly to generate the plasmid pYXZ-ySOD1. To express human *SOD1*, the yeast *SOD1* coding region in pYXZ-ySOD1 was replaced with the human *SOD1* ORF, yielding pYXZ-hSOD1. Mutant *SOD1* alleles were constructed using site-directed mutagenesis; briefly, two overlapping fragments containing the target mutations were amplified from pYXZ-ySOD1 or pYXZ-hSOD1 and subsequently re-assembled into the backbone using Gibson assembly. All primers used for plasmid construction are listed in S2 Table.

### Detection of Rad52 foci

To visualize Rad52 foci, wild-type and sod1 mutant strains expressing YFP-tagged Rad52 were cultured in YPD medium to log phase (OD_600_ = 0.1). High-resolution fluorescence imaging was performed using an Olympus FV3000 confocal microscope (Japan). Rad52-YFP foci were quantified from Z-stack images [58], with data representing the mean of three independent biological replicates.

## Supporting information

S1 Text Strain construction. (DOCX)

S1 Table Yeast strains used in this study. (XLSX)

S2 Table Primers used in this study. (XLSX)

S1 Fig Effects of *SOD1* deletion on the phenotype and mutation rate of a bioethanol yeast derivative. **(A)** Deletion of *SOD1* impairs the growth of strain YJSHa (a haploid derivative of YJS329) under both standard and oxidative stress conditions. **(B)** Deletion of *SOD1* significantly elevates mutation rates, as measured by the *URA3* forward mutation assay. Experiments were performed by three times. Statistical significance is indicated as ***P < 0.001. *P* values were calculated using the Wilcoxon rank-sum test.
(TIF)

S1 Data Genomic location of LOH events in 39 isolates of *sod1* strain. (XLSX)

S2 Data Depictions of various classes of LOH events. (XLSX)

S3 Data Chromosomal rearrangements detected in the *sod1* isolates. (XLSX)

S4 Data Point mutations detected in the *sod1* isolates. (XLSX)

S5 Data Summary of sectored colonies in WYsod1. (XLSX)

S6 Data Associations of LOH events with various elements of chromosome structure. (XLSX)

S7 Data Aneuploidy events in the *sod1* isolates. (XLSX)
