# Supplementary material for: Decoupling metabolic defense: Mutational and recombinational chaos in Sod1-deficient cells
Source: PLoS Genet. 2026 Oct 5;22(10):e1012326. doi: 10.1371/journal.pgen.1012326 (PMC13649116; doi:10.1371/journal.pgen.1012326)
Supplement: Supplementary file 1 [file pgen.1012326.s001.docx]

**S1 Text. Strain construction.**

The genotypes and brief construction description of the strains used in this study are in S1 Table. The primers used for strain construction are listed in S2 Table. Strain JSC25 is a diploid strain generated by crossing two haploid strains (W303-1A and YJM789) in [1]. This strain was used to determine the rate of spontaneous crossover events on chromosome IV. The deletion of *SOD1* genes in SY165 and SY166 [2] resulted in Wsod1 and Ysod1 respectively (S1 Table). The deletion cassette was amplified by one-step PCR using the selection mark containing plasmid (pAG32 and pUG72) as a template with primers dSOD1S and dSOD1A (S2 Table). Crossing of Wsod1 and Ysod1 generates the diploid *sod1* mutant, WYsod1. To generate a W303-1A background strain carrying the wild-type *SSD1* allele (named as SY21-1), the nonsense ssd1 gene in the W303-1A strain was initially replaced with *URA3*, which was subsequently replaced by wild-type *SSD1* cassettes. Transformants containing *SSD1* were screened using synthetic complete medium supplemented with 1.1g/L 5-FOA. The *ogg1*, *rev1*, *rev3*, and *rad30* mutants were constructed by transformation of deletion cassette amplified from plasmid pAG25. The details of construction are shown in S1 Table.

**Supplemental references**

1. St Charles J, Petes TD. High-resolution mapping of spontaneous mitotic recombination hotspots on the 1.1 Mb arm of yeast chromosome IV. PLoS Genet. 2013;9:e1003434.
2. Sui Y, et al. Genome-wide mapping of spontaneous genetic alterations in diploid yeast cells. Proc Natl Acad Sci U S A. 2020;117:28191-28200.
3. Song W, Dominska M, Greenwell PW, Petes TD. Genome-wide high-resolution mapping of chromosome fragile sites in *Saccharomyces cerevisiae*. Proc Natl Acad Sci U S A. 2014;111:E2210-E2218.
4. Zheng DQ, et al. Genome sequencing and genetic breeding of a bioethanol *Saccharomyces cerevisiae* strain YJS329. BMC Genomics. 2012;13:479.
